# IT’S OVERWHELMING FOR EVERYBODY: EXPERIENCES OF DEMENTIA CAREGIVERS WHO AREN’T CLOSE FAMILY

**DOI:** 10.1093/geroni/igad104.1220

**Published:** 2023-12-21

**Authors:** Jane Lowers, Ivree Datcher, Michelle Delk, Dio Kavalieratos, Molly Perkins, Kenneth Hepburn

**Affiliations:** Emory School of Medicine, Atlanta, Georgia, United States; Emory University, Atlanta, Georgia, United States; Emory Rol, Atlanta, Georgia, United States; Emory University, Atlanta, Georgia, United States; Emory University, Atlanta, Georgia, United States; Emory University, Atlanta, Georgia, United States

## Abstract

One in three people with Alzheimer’s or other dementias lives alone, without a spouse/partner or nearby children (i.e., is aging solo). Friends, neighbors, and extended family often assist with tasks that preserve independence, but most dementia caregiving literature, and theory, to date focuses on spousal or parent/child dyads for whom reciprocity, indebtedness and self-interest are common motivators. This study seeks to address this knowledge gap. Design: Semi-structured interviews with caregivers of adults with cognitive impairment or dementia who were not the caregivers’ spouses or parents. Hybrid inductive/deductive thematic analysis incorporating social exchange and stress-coping theories.

Results: We interviewed 11 caregivers (100% female; age 54-85, mean 71; 91% white, 9% black; 27% friend; 27% church congregant; 27% sibling or in-law; 18% neighbor). Caregivers described altruism, empathy, and, in cases of extended family, duty as motivators to provide care for acquaintances with dementia. Caregivers who themselves lacked close family identified with their care recipient’s vulnerability and the possibility of needing similar care someday. Non-family caregivers described feeling unprepared and unqualified to make legal, financial, and guardianship decisions and looked to care recipients’ distant family, health care providers, and religious leaders to guide decisions that require balancing care recipients’ safety and autonomy, such as moving to a nursing home.

Conclusion: Dementia caregiving outside spouse or parent/child dyads can produce practical and emotional challenges for caregivers who are not immediate family. These caregivers may benefit from guidance about practical and ethical decisions in the absence of immediate family decision makers.

